# Porphyran from *Porphyra haitanensis* Enhances Intestinal Barrier Function and Regulates Gut Microbiota Composition

**DOI:** 10.3390/md21050265

**Published:** 2023-04-26

**Authors:** Sathuvan Malairaj, Suresh Veeraperumal, Wanzi Yao, Mugesh Subramanian, Karsoon Tan, Saiyi Zhong, Kit-Leong Cheong

**Affiliations:** 1 Guangdong Provincial Engineering Technology Research Center of Seafood, Guangdong Provincial Science and Technology Innovation Center for Subtropical Fruit and Vegetable Processing, Guangdong Provincial Key Laboratory of Aquatic Product Processing and Safety, Guangdong Province Engineering Laboratory for Marine Biological Products, College of Food Science and Technology, Guangdong Ocean University, Zhanjiang 524088, China; 2 Department of Biology, College of Science, Shantou University, Shantou 515063, China; 3 School of Food Science and Engineering, South China University of Technology, Guangzhou 510640, China; 4 Research and Development Center, Genexia Bioserv, Chennai 600045, Tamilnadu, India; 5 Guangxi Key Laboratory of Beibu Gulf Biodiversity Conservation, Beibu Gulf University, Qinzhou 535011, China; tankarsoon@bbgu.edu.cn

**Keywords:** *Porphyra haitanensis*, porphyran, intestinal barrier, gut microbiota

## Abstract

In this study, the effects of a homogenous porphyran from *Porphyra haitanensis* (PHP) on the intestinal barrier and gut microbiota were investigated. The results showed that oral administration of PHP resulted in a higher luminal moisture content and a lower pH environment for the growth of beneficial bacteria in the colon of mice. PHP significantly increased the production of total short-chain fatty acids during the fermentation process. PHP made the intestinal epithelial cells of mice arrange more tidily and tightly with a significant increase in mucosal thickness. PHP also increased the amount of mucin-producing goblet cells and the expression of mucin in the colon, which maintained the structure and function of the intestinal mucosal barrier. Moreover, PHP up-regulated the expression of tight junctions including ZO-1 and occludin, improving the intestinal physical barrier function. The results of 16S rRNA sequencing showed that PHP regulated the composition of gut microbiota in mice, increasing the richness and diversity of gut microbiota and the ratio of Firmicutes to Bacteroidetes. This study revealed that the intake of PHP is beneficial for the gastrointestinal tract and PHP could be a potential source of prebiotics in the functional food and pharmaceutical industries.

## 1. Introduction

The human gastrointestinal tract is home to trillions of microorganisms, collectively referred to as the gut microbiota, which play a crucial role in maintaining host health. The gut microbiota plays a crucial role in numerous vital physiological processes such as nutrient metabolism, immune system development, and maintenance of the intestinal epithelial barrier function [1]. The intestinal barrier is a complex system that protects the gut from harmful substances while allowing the absorption of nutrients. The barrier is composed of several layers, including the mucus layer and the epithelial layer, which is made up of tight junctions between cells [2]. The intestinal barrier is made up of various cell types, including enterocytes, goblet cells, Paneth cells, and immune cells. Enterocytes are the most abundant cells in the intestinal epithelium and act as the primary physical barrier between the gut lumen and underlying tissue [3]. Goblet cells produce mucus that protects and lubricates the intestinal epithelium, while Paneth cells secrete antimicrobial peptides to maintain a healthy gut microbiome. Immune cells are essential for detecting and eliminating harmful substances that may compromise the integrity of the intestinal barrier [3]. Disruption of the intestinal barrier can lead to increased inflammation and bacterial translocation, contributing to various gastrointestinal disorders. Disruption of the gut microbiota has also been linked to various diseases, including colorectal cancer, inflammatory bowel disease, ulcerative colitis obesity, and type 2 diabetes [4]. Therefore, maintaining the function of the intestinal barrier and the healthy balance of gut microbiota is crucial for overall health.

Dietary factors are known to have a significant impact on the composition and function of the intestinal microbiota and the integrity of barrier function. Prebiotics, which are food ingredients that are non-digestible and selectively stimulate the growth and/or activity of beneficial gut bacteria, while also enhancing the function of the intestinal barrier, have gained increasing attention as potential functional food ingredients with health-promoting effects [5,6]. Among various prebiotics, polysaccharides derived from seaweed have been reported to have beneficial effects on gut microbiota and host health [7,8,9,10]. Polysaccharides will be depolymerized into simpler compounds in the gastrointestinal tract, which can be used by the intestinal microbiota. Bacteria can convert complex organic compounds, such as short-chain fatty acids (SCFAs), into simpler compounds through various metabolic pathways [11]. The production of a broad range of metabolites with significant effects on host health is a result of the different metabolic capabilities of bacterial species and their interactions [11]. The breakdown of metabolites by bacteria is critical for maintaining microbial communities and nutrient cycling in the environment. Seaweed-derived sulfated polysaccharides provide prebiotic activity by enhancing the energy metabolism of gut microbiota and regulating gut microbial hemostasis [12,13]. In addition, they safeguard the integrity of the intestinal mucosal barrier function by promoting the development and functional maturation of epithelial cells and mucosal immune cells, while also stimulating the secretion of mucus by goblet cells [14]. The physicochemical properties of seaweed polysaccharides, such as chemical structure, molecular weight, solubility, and water retention quality, can affect their physiological effects on the intestine [15].

*Porphyra haitanensis*, a type of red seaweed commonly consumed in Asian countries, contains large amounts of bioactive polysaccharides. Porphyran, a sulfated polysaccharide rich in *P. haitanensis*, has been reported to have various biological properties, such as antioxidant [16], anticancer [17,18], anti-inflammatory [19], anti-obesity [20], and anti-photoaging [21] effects. Our research group extracted and purified a homogenous porphyran from *P. haitanensis* (named PHP), which was composed of a repeating structure made up of alternating (1→4)-linked 3,6-anhydro-α-l-galactopyranose residues or (1→4)-linked α-l-galactose 6 sulfate residues with a molecular weight of 2.01 × 10^5^ Da [22]. In vitro studies have shown that PHP has positive effects on intestinal epithelial cells, as it can enhance the healing of intestinal wounds by promoting the proliferation and migration of cells [22]. Furthermore, in vitro fermentation studies have shown that PHP possessed potential prebiotic effects as it reshaped the structure of intestinal microbiota by promoting the growth of intestinal probiotics and suppressing that of harmful bacterial communities [23]. However, all our previous studies were in vitro [22,23]. The in vivo fermentation behavior of PHP and its effects on the intestinal barrier and intestinal microbiota in vivo remains unknown. In vivo studies are necessary to fully understand the potential effects of PHP on the microbiota flora and intestinal barrier in a whole organism, taking into account the complexities of the physiological and metabolic processes that occur in vivo.

Therefore, this study aimed to assess how PHP affects the functioning of the intestinal barrier and microbiota composition in mice. The in vivo fermentation processes are responsible for regulating the pH levels and moisture content of colon luminal contents, colon length, and colon index, and the composition and concentration of SCFAs were evaluated. The effects of PHP on gut health were assessed by analyzing the intestinal physiological conditions of mice and the expression of mucin, tight junctions, and myeloperoxidase (MPO). The fecal microbiota composition was also analyzed using 16S rRNA sequencing technology. This study will pave the way for the application of seaweed polysaccharides with vital intestinal health-promoting functions.

## 2. Results and Discussion

### 2.1. Fourier Transform Infrared Spectroscopy (FT-IR) Analysis

**Figure 1:**
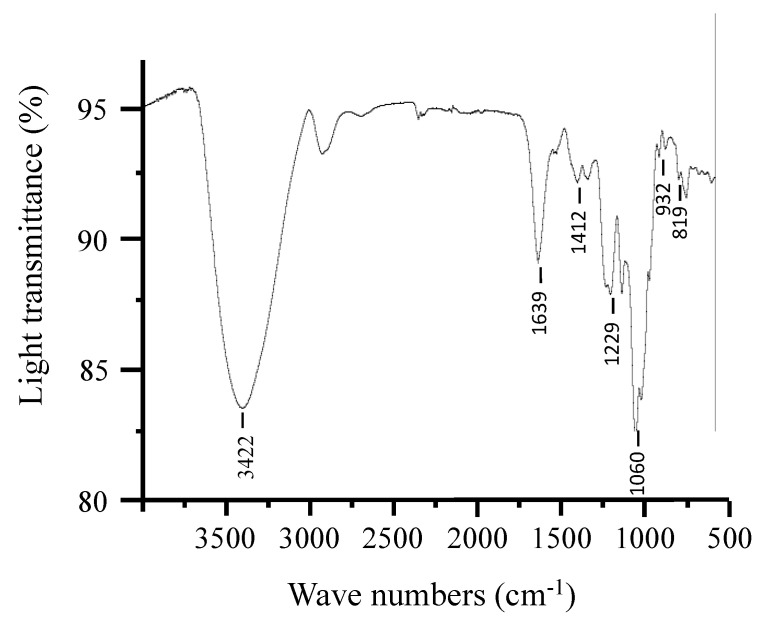
FT-IR spectrum of PHP.

Our previous research showed that PHP is composed of a repeating structure of alternating (1→4)-linked 3,6-anhydro-α-L-galactopyranose units or (1→4)-linked α-l-galactose 6 sulfate units [22]. In this study, the structure of purified PHP was analyzed to reconfirm whether different batches of extraction and purification processes make difference in structural changes in PHP or not. The sugar composition of PHP was analyzed using GC-MS, revealing a ratio of 1.2:1.0 for galactose and 3,6-anhydrogalactose. Additionally, the total sulfate content of PHP was found to be 5.8% ± 0.42. PHP has a molecular weight of 2.01 × 10^5^ Da. The analysis of the structure of polysaccharides, including functional groups and glycosidic bonds, can be effectively carried out using FT-IR, which is a reliable technique. The FT-IR spectrum of PHP is shown in Figure 1. The results revealed that PHP had typical signals of porphyran, such as at 3422, 1639, 1412, 1229, 1060, 932, and 819 cm^−1^ [24]. The weak band 932 cm^−1^ was attributed to the absorption of 3,6-anhydrogalactose and 819 cm^−1^ was assigned to the absorption of the sulfate group attached at the C-6 position of galactose [25]. The results are consistent with our previous study.

### 2.2. Effects of PHP on the Intestinal Physiological Status

**Figure 2:**
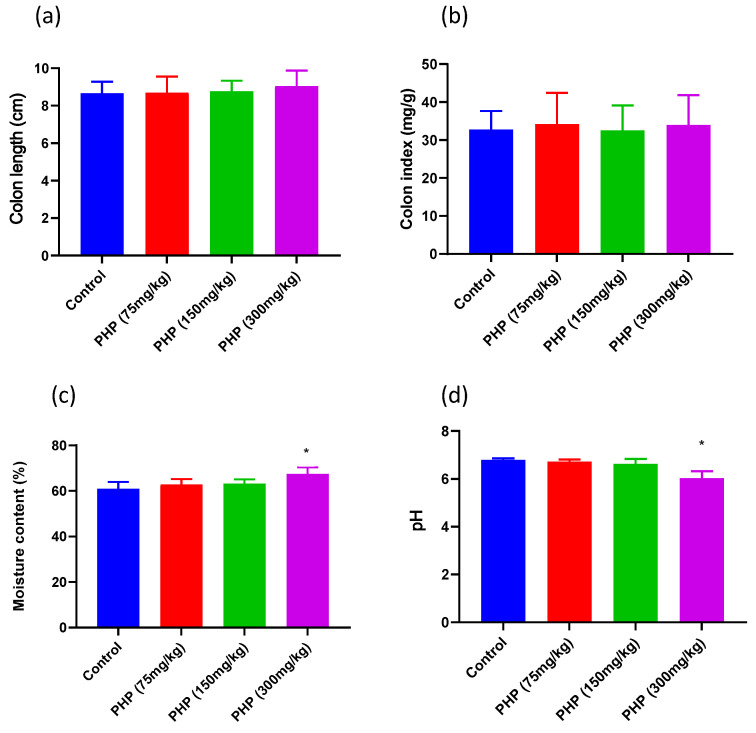
Effects of PHP on intestinal physiological status in mice (*n* = 6). (**a**) Colon length, (**b**) colon index, (**c**) moisture content, and (**d**) pH value. Values are expressed as mean ± SD. * *p* < 0.05 vs. Control group.

The intestinal mucosal barrier is a critical component of the intestinal physiological status and plays a vital role in maintaining intestinal health [26]. The intestinal barrier function can be reflected by intestinal growth parameters such as colon length, colon weight, and colon environment parameters. The growth and development of intestinal cells and tissues are important for maintaining the integrity of the intestinal barrier. The presence of healthy gut microbiota, which can be influenced by luminal environment parameters such as pH and moisture content, also plays an essential role in promoting a healthy intestinal barrier [27]. As shown in Figure 2a, the colon lengths and colon indexes of mice treated with different doses of PHP were not significantly increased when compared to the control. However, the colon moisture contents of mice treated with 300 mg/kg of PHP were significantly higher than those in the control group (*p* < 0.05). This suggested that PHP administration produced a stronger water-holding capacity for the intestinal luminal content, which could result in the reduction of defecation time [28]. Moreover, the colon pH values of mice in the PHP (300 mg/kg) group significantly decreased when compared to the control group (*p* < 0.05). This may be due to the PHP fermentation products from the microbiota in the gastrointestinal tracts [29]. Studies have shown that maintaining a lower pH value and higher moisture content in the gut environment can help to promote gut health and reduce the risk of developing intestinal diseases [11]. Therefore, the changes observed in the physiological status of the intestine, specifically the increase in luminal moisture content and decrease in luminal pH value following PHP administration, can be considered beneficial for maintaining gut health. In addition, the rise in intestinal luminal moisture facilitated the dissolution of organic acids, mucins, and immune factors that are essential for maintaining intestinal homeostasis. This indicates that the improved physiological status resulting from PHP administration is closely linked to the functioning of the intestinal barrier. Hence, further study was subjected to analyze the organic acids, intestinal barrier function, and mucin secretion in mice.

### 2.3. Effects of PHP on the Production of SCFAs

**Figure 3:**
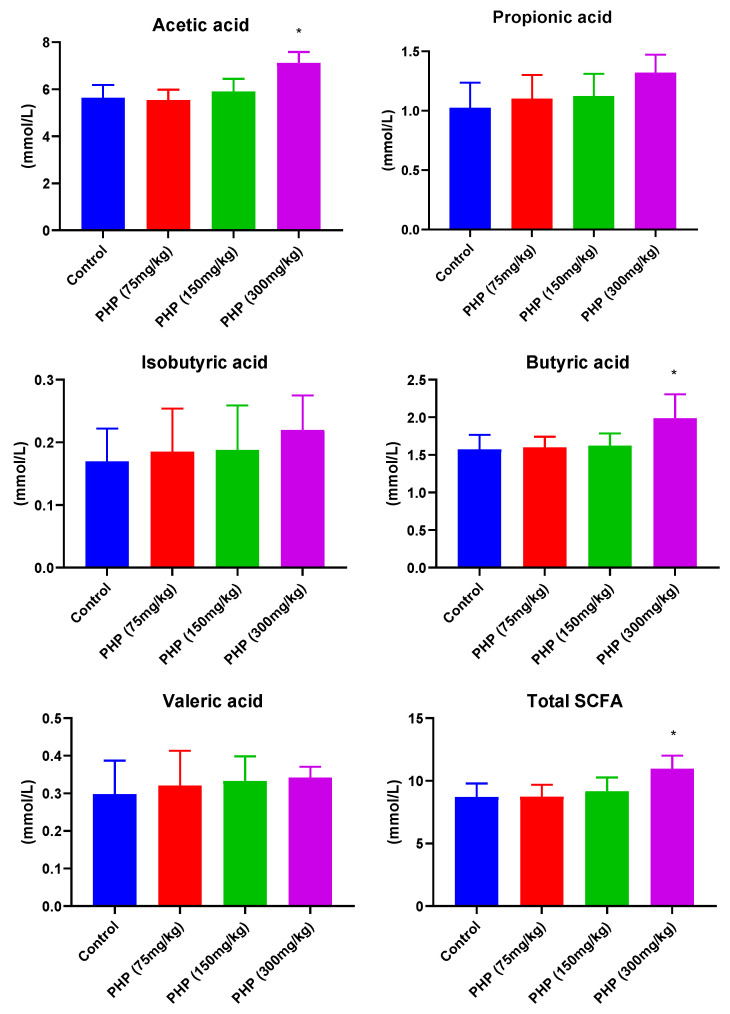
Effects of PHP on the concentration of SCFAs in the colon luminal contents of mice (*n* = 6). Values are expressed as mean ± SD. * *p* < 0.05 vs. Control group.

SCFAs, consisting of a carboxylic acid moiety and hydrocarbon chain, are produced by the fermentation of non-digestible polysaccharides by gut microbiota. SCFAs exhibit a diverse range of pharmacological effects, including the regulation of lipid metabolism, modulation of the immune system, and maintenance of gut integrity and homeostasis [30]. According to the results presented in Figure 3, the concentration of total SCFAs in the colon of mice treated with 300 mg/kg of PHP was significantly greater compared to that of the control group (*p* < 0.05). The higher concentration of total SCFAs helped to regulate the pH of the colon, promoting a slightly acidic environment that inhibited the growth of harmful bacteria while supporting that of beneficial bacteria. Specifically, the concentrations of acetic acid and butyric acid were found to be significantly higher in the PHP (300 mg/kg) group compared to the control group (*p* < 0.05). Acetic acid is the most abundant SCFA in the gut, accounting for approximately 60–70% of the total SCFA content [31]. It is produced by many different types of gut bacteria, including Bacteroidetes, Firmicutes, and Actinobacteria. Butyric acid is the third most abundant SCFA in the gut, accounting for approximately 10–15% of the total SCFA content. It is mainly produced by firmicutes bacteria, such as *Clostridia*. These bacteria metabolize the polysaccharides using enzymes to break down the carbohydrate molecules into smaller sugars, such as glucose and fructose [14]. The bacteria then convert these simple sugars into SCFAs, including acetic acid, through a process known as glycolysis. SCFAs serve as an energy source for colonocytes, the cells that line the colon, helping to maintain their health and function [32]. They also contribute to the integrity of the gut barrier, helping to prevent the leakage of harmful substances from the gut into the bloodstream. The consumption of PHP significantly increased the generation of total SCFAs during its digestion and metabolism in the intestinal tract in the intestinal tract, enhancing the function of the intestinal barrier. Furthermore, the low pH value of the intestine could also play a helpful role in the production of SCFAs. The intake of PHP decreased the pH value of colon contents, which was essential to produce beneficial intestinal metabolites such as SCFAs.

### 2.4. Effects of PHP on the Intestinal Mucosal Barrier

**Figure 4:**
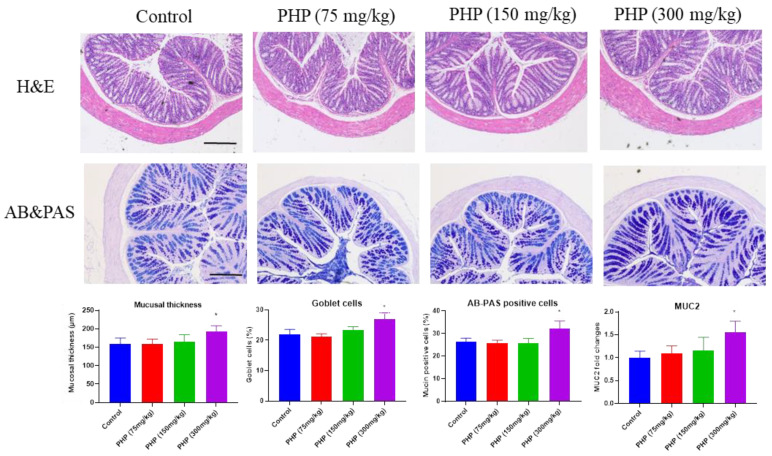
Histological analysis of mice’s colon, including H&E staining and AB&PAS staining (*n* = 6). Bars = 100 µm. Values are expressed as mean ± SD. * *p* < 0.05 vs. Control group.

The pathological morphology of the colon was evaluated using hematoxylin and eosin (H&E) staining or Alcian blue and periodic acid Schiff (AB&PAS) staining. The H&E staining analysis in Figure 4 reveals notable differences in the morphological structures of the intestinal mucosa between the different groups following the oral administration of PHP. The colon tissue in the PHP (300 mg/kg) group was well-organized, with identifiable crypt structures, intact mucosa, and abundant goblet cells that continuously secreted mucus, forming and maintaining a protective barrier in response to stimulation.

AB&PAS staining is a laboratory technique used to detect and evaluate the presence of mucin-producing cells and mucin deposition in tissues. Mucins are glycoproteins that are present in various tissues throughout the body, including the respiratory and gastrointestinal tracts [33]. In the gastrointestinal tract, mucins form a protective barrier between the epithelial cells that line the intestinal wall and the potentially harmful contents of the gut, such as digestive enzymes and microorganisms. Mucins are produced and secreted by goblet cells, which are found in the epithelial lining of various tissues. As shown in Figure 4, the proportion of goblet cells in the PHP (300 mg/kg) group was considerably greater compared to the control group (*p* < 0.05). Moreover, the AB-PAS staining demonstrated a greater area of positive staining for mucins in the group receiving PHP (300 mg/kg) compared to the control group.

**Figure 5:**
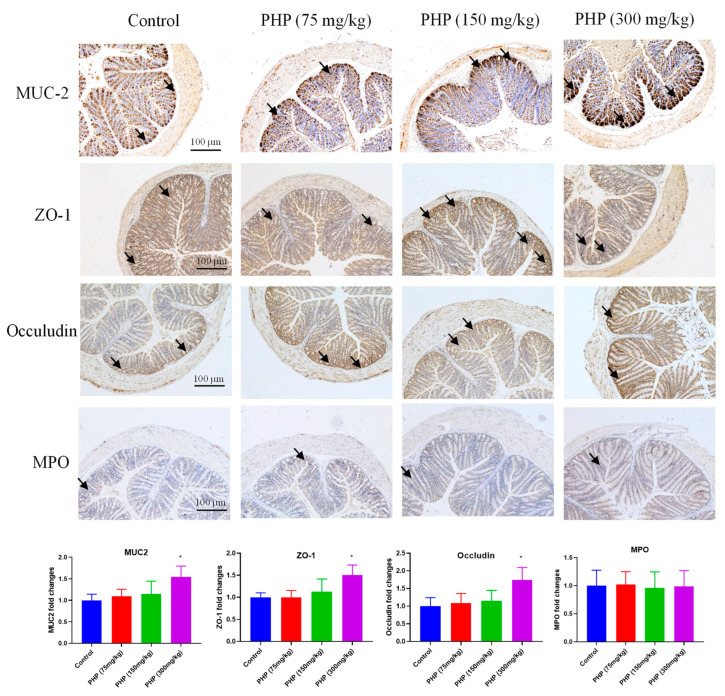
Immunohistochemical analysis of MUC-2, ZO-1, Occludin, and MPO in mice’s colon (*n* = 6). The arrows indicate the MUC-2, ZO-1, Occludin, and MPO, respectively. Bars = 100 µm. Values are expressed as mean ± SD. * *p* < 0.05 vs. Control group.

Mucin-2 (MUC-2) plays a significant role in organizing the intestinal mucus layers as a crucial member of the mucin family. It is the most abundant mucin secreted in the intestine and is primarily produced by goblet cells in the colonic epithelium [34]. MUC-2 plays a critical role in forming the backbone of the colonic mucus layer, serving as its major structural component. The deficiency of MUC-2 can persuade the harmful microorganisms to interface with intestinal epithelial cells directly and consequently progress unprompted colitis. The expression of MUC-2 was investigated by immunohistochemistry. As shown in Figure 5, oral administration of PHP at a high dose (300 mg/kg) significantly up-regulated the expression of MUC-2 (*p* < 0.05). The results suggested PHP promoted mucin synthesis in the intestine and fortify the intestinal mucus layer. Intestinal mucin production and epithelial integrity play a substantial role in intestinal barrier functions. Therefore, PHP increased the amount of mucin-producing goblet cells and the expression of mucin in the colon, which maintained the structure and function of the intestinal mucosal barrier.

### 2.5. Effects of PHP on the Expression of Tight Junctions and MPO

In addition to the physical layers, the intestinal barrier also includes tight junctions between enterocytes. The preservation of cellular tight junctions among intestinal epithelial cells is crucial for maintaining mucosal integrity, which is essential for intestinal barrier function [35]. ZO-1 and occludin play a critical role in regulating the movement of substances across the intestinal epithelium [36]. They function as a discerning barricade to impede the transit of noxious substances, such as bacteria and toxins, from the lumen of the intestine into the bloodstream. They also help to regulate the absorption of nutrients and water by allowing only certain substances to pass through the intestinal epithelium [37]. The disruption of tight junctions may raise the penetrability of the intestinal epithelium, cause damage to the structure of the intestinal mucosa, and lead to bacterial translocation as a result of the increased level of endotoxin in the serum [38]. This has been linked to a variety of health conditions, including inflammatory bowel disease, celiac disease, and autoimmune disorders. The expression of ZO-1 and occludin was investigated by immunohistochemistry. As shown in Figure 5, the expression of ZO-1 and occludin in the colon significantly increased by PHP (300 mg/kg) when compared to the control. This revealed PHP fortified the tight junctions of intestinal epithelial cells to up-regulate the intestinal integrity and improve the intestinal mucosal barrier function, which was supplementarily sustained by promoting the development of intestinal mucosal morphology (Figure 3).

The intestinal barrier also contains immune cells that help to identify and neutralize potential threats [39]. Neutrophils are a type of immune cell that plays a critical role in fighting bacterial and fungal infections [40]. However, excessive neutrophil infiltration can also be harmful and contribute to tissue damage and inflammation. MPO is an enzyme released by neutrophils and helps to destroy invading microorganisms by producing hypochlorous acid and other free radicals [41]. These compounds are toxic to bacteria and fungi and can help to kill them. However, excessive MPO activity can also contribute to tissue damage and inflammation. In some inflammatory conditions, such as atherosclerosis, asthma, and inflammatory bowel disease, MPO has been shown to promote oxidative stress and inflammation, leading to tissue damage. Therefore, the role of MPO in the intestinal barrier is complex and context-dependent. Maintaining a balanced and controlled immune response is crucial for the maintenance of the intestinal barrier and the prevention of inflammatory diseases. The expression of MPO was evaluated by immunohistochemistry. However, the PHP (300 mg/kg) group demonstrated no significant difference in the expression of MPO compared to the control group (Figure 5).

### 2.6. Effects of PHP on the Gut Microbiota

**Figure 6:**
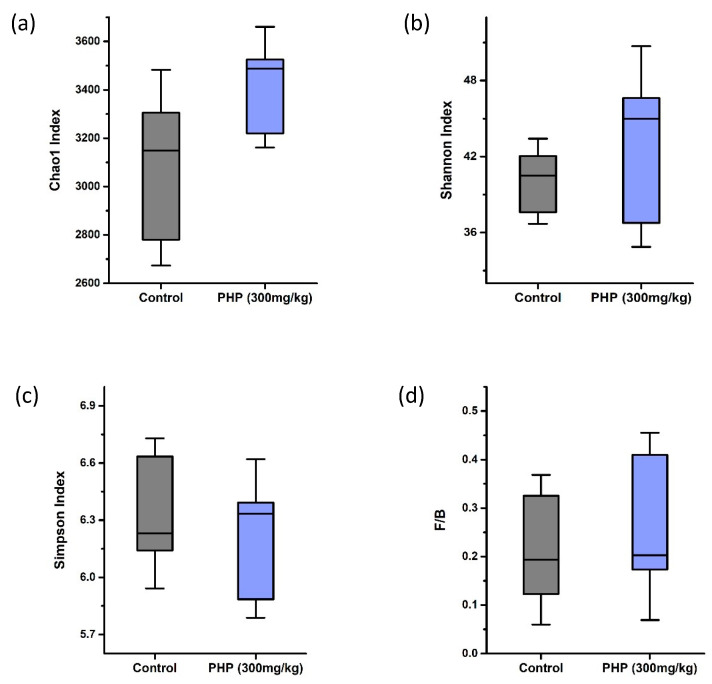
Alpha diversity analysis of gut microbiota (*n* = 6). (**a**) Chao1 index, (**b**) Shannon index, and (**c**) Simpson index. (**d**) The ratio of Firmicutes to Bacteroidetes (F/B ratio). Values are expressed as mean ± SD.

Seaweed polysaccharides cannot be digested by intestinal enzymes, which permit them to reach the large intestine to selectively ferment by intestinal microbes, consequently impelling the nutrition absorption, energy spending, and immune system of the host [42]. In this study, the regulatory effects of PHP on intestinal microbiota were evaluated through metagenomic analyses by 16S rRNA sequencing. Alpha diversity is an important measure of the diversity and complexity of the microbial communities within the gut. A high level of alpha diversity within the gut microbiota is generally considered to be a marker of a healthy gut, as it indicates a more diverse and stable microbial community with a greater number of species able to survive and thrive within the gut environment. There are several different ways to measure the alpha diversity of gut microbiota, including the calculation of diversity indices such as the Chao1 index, Shannon index, and Simpson index. The Chao1 index is a commonly used metric for estimating the species richness of microbial communities, including the gut microbiota. The Shannon index and the Simpson index are two commonly used diversity indices for measuring the diversity of microbiota. The Shannon index takes into account both the number of different species present in a microbial community and the relative abundance of each species. The Simpson index, on the other hand, is a measure of the dominance of a few highly abundant species in a community. As shown in Figure 6a,b, the Chao1 index and Shannon index in the PHP (300 mg/kg) group were higher than those in the control group. This suggested that the intake of PHP increased the richness and diversity of gut microbiota in mice.

**Figure 7:**
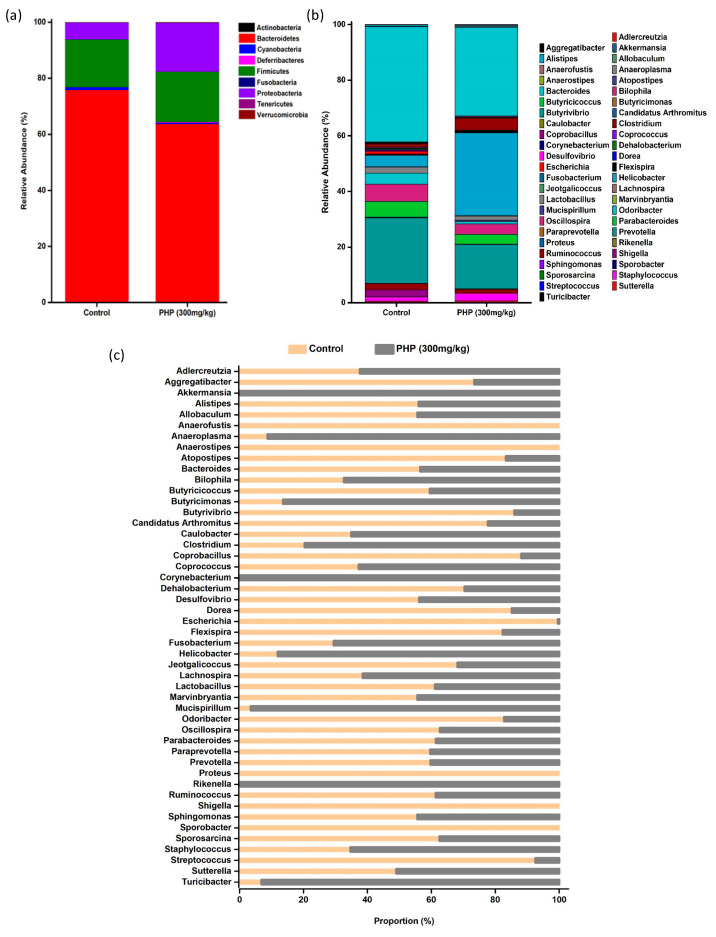
The composition of gut microbiota in mice at the (**a**) phylum level and (**b**) genus level (*n* = 6). (**c**) The relative abundance proportion of gut microbiota at the genus level. Values are expressed as mean ± SD.

The composition of intestinal flora in mice at the phylum level is shown in Figure 7a. Nine distinct phyla were detected. Among them, Bacteroidetes, Firmicutes, and Proteobacteria are predominant, while Actinobacteria, Cyanobacteria, Deferribacteres, fusobacteria, tenericutes, and verrucomicrobia are less abundant. The Firmicutes to Bacteroidetes ratio (F/B ratio) is a commonly used metric for assessing the composition of the gut microbiota. Firmicutes and Bacteroidetes are two of the most prevalent bacterial phyla identified in the human gut, and their relative abundance can be used to provide insight into the overall composition and potential function of the microbiota. A higher F/B ratio has been linked to increased production of SCFAs, which can have anti-inflammatory effects and may play a role in maintaining gut health [43]. As shown in Figure 7a, the relative abundance of firmicutes in the control and PHP-treated groups showed similarly, and no significant changes were observed, whereas the relative abundance of Bacteroidetes in the PHP-treated group decreased compared to the control. Therefore, the F/B ratio in the PHP (300 mg/kg) group was lower than that in the control group (Figure 6d). Other than Firmicutes and Bacteroidetes, a noticeably increased relative abundance of Proteobacteria was observed in the PHP (300 mg/kg) group. Some strains of Proteobacteria also have been shown to produce antimicrobial compounds that can inhibit the growth of pathogenic bacteria, therefore helping to maintain the balance of the gut microbiota [44]. Moreover, a slight decrease in the abundance of Cyanobacteria was observed in the PHP-treated group. Studies have shown that Cyanobacteria can interact with intestinal epithelial cells and disrupt the integrity of the gut barrier. A decrease in the abundance of Cyanobacteria can therefore help to improve gut barrier function and reduce the risk of leaky-gut syndrome [45].

The composition of intestinal flora in mice at the genus level is shown in Figure 7b and its relative abundance proportion between the two groups is shown in Figure 7c. Some genera such as *Anaerofustis*, *Proteus*, *Shigella*, and *Sporobacter* were observed in the control group but not found in the PHP-treated group. *Proteus* spp. are initially considered low-abundance commensals of the gut, but recent correlation with Crohn’s disease revealed its pathogenic potential [46]. *Shigella* is a well-known pathogen that can cause intestinal inflammation and infection, which can lead to damage to the intestinal lining, disruption of the intestinal flora, and an increased risk of developing other gastrointestinal diseases [47]. *Akkermansia*, *Corynebacterium*, and *Rikenella* were observed in the PHP-treated group but not in the control group. *Akkermansia* has been shown to enhance the production of mucus in the gut, which helps to protect the intestinal lining from harmful substances and pathogens. It has also been found to increase the expression of tight junction proteins, which help to maintain the integrity of the intestinal barrier [48]. *Rikenella* is negatively correlated with markers of intestinal inflammation and positively correlated with markers of intestinal barrier function, indicating its potential protective role against intestinal mucosal damage [49]. The abundance of *Aggregatibacter*, *Alistipes*, *Allobaculum*, *Atopostipes*, *Bacteroides*, *Butyricicoccus*, *Butyrivibrio*, *Candidatus*, *Coprobacillus*, *Dehalobacterium*, *Desulfovibrio*, *Dorea*, *Escherichia*, *Flexispira*, *Jeotgalicoccus*, *Lactobacillus*, *Odoribacter*, *Oscillospira*, *Parabacteroides*, *Paraprevotella*, *Prevotella*, *Ruminicoccus*, *Sporosarcina*, and *Streptococcus* in the PHP (300 mg/kg) group was lower than that in the control group. In addition, the abundance of *Allercreutzia*, *Anaeroplasma*, *Bilophila*, *Butyricimonas*, *Caulobacter*, *Clostridium*, *Coprococcus*, *Fusobacterium*, *Helicobacter*, *Lachnospira*, *Mucispirillum*, *Staphylococcus*, and *Turicibacter* in the PHP-treated group was higher compared to the control. The genus of *Lachnospira*, *Lachnoclostridium*, *Fusicantenibacter*, *Roseburia*, *Eubacterium*, *Ruminococcus*, and some genera from *Bacteroidetes* are the major butyrate producer for gut mucosal immune regulation and functional marker of healthy mature anaerobic gut microbiota [50]. *Coprococcus* plays a significant role in the production of vitamin B and SCFAs [51,52]. The *Faecalibacterium prausnitzii*, *Roseburia intestinalis*, and *Agathobacter rentals* are the common bacteria known to produce butyrate [53].

**Figure 8:**
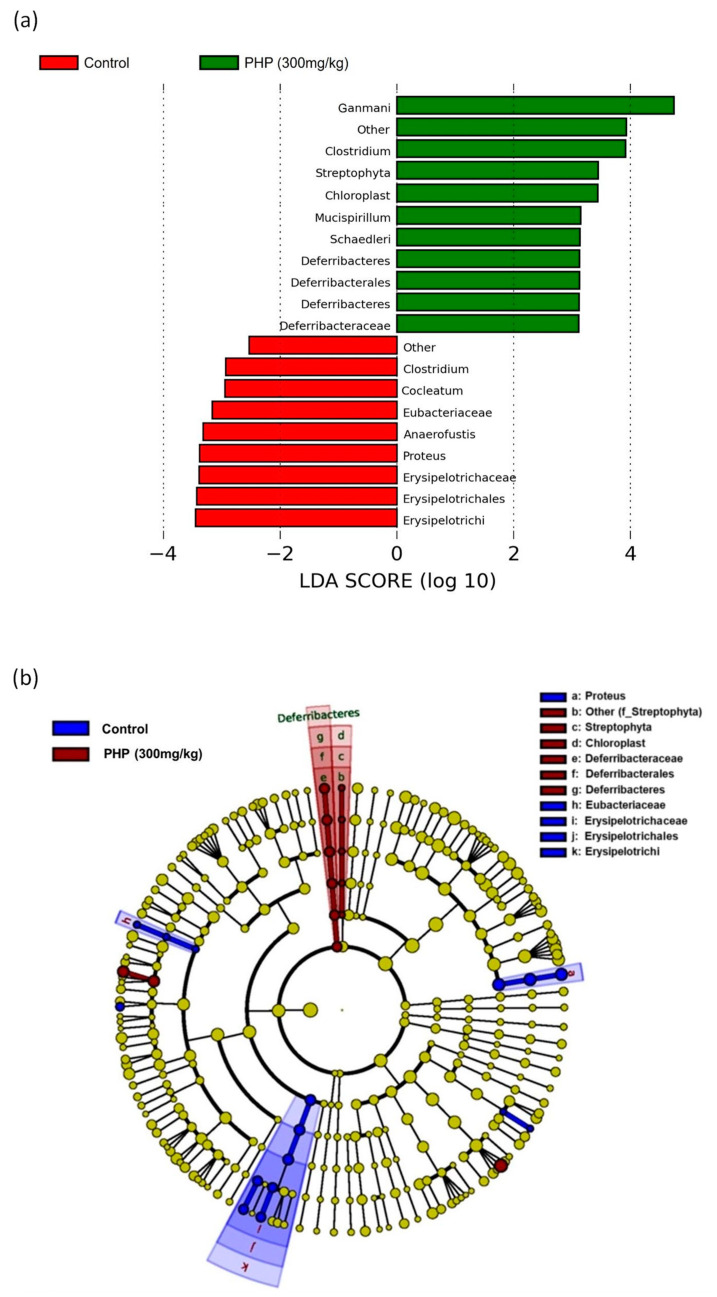
LefSe analysis of the predominant taxa of gut microbiota (*n* = 6). (**a**) Logarithmic LDA score and (**b**) cladogram representation of LDA score. Values are expressed as mean ± SD.

Linear discriminant analysis effect size (LefSe) is a statistical method used to identify the features that are differentially abundant between two or more groups. It is commonly used in microbiome studies to identify biomarkers or taxa that are associated with specific clinical or environmental conditions. The output of LefSe includes the effect size, which measures the strength and direction of the difference between groups, and the LDA score, which is a measure of the statistical significance of the effect size. As shown in Figure 8, there were significant differences between the groups’ predominant taxa of intestinal microflorae by setting 4.0 as the LDA score. Among them, the class of *Deferribacteres* was found to be dominant in the PHP group, which was one of the main bacterial groups responsible to produce butyrate in the human colon [54]. In contrast, the class of Erysipelotrichia was dominant in the control group, which has been implicated in the development of inflammatory bowel disease and other gut-related disorders [55]. Moreover, the *Clostridium* genus was found to be common in both control and PHP-treated groups. Some strains of Clostridium are beneficial, helping to ferment dietary fibers and produce beneficial SCFAs [56].

## 3. Material and Methods

### 3.1. Materials and Chemicals

*P. haitanensis* was collected from Nan’ao Island, Shantou, Guangdong Province, China. Acetic, propionic, isobutyric, butyric, and valeric are among the standard SCFAs that were acquired from Aladdin Chemical Co. (Shanghai, China). All other reagents and chemicals used were of analytical grade.

### 3.2. Preparation and Characterization of PHP

PHP was prepared from *P. haitanensis* according to our previous study [16]. Its physicochemical property was studied through FT-IR analysis. FT-IR analysis was performed on an FT-IR spectrophotometer (MAGNA-IR 750, Thermo Nicolet Co., Madison, WI, USA). The PHP sample was processed by grinding with potassium bromide, followed by pellet pressing and detection within the wavelength range of 4000–500 cm^−1^.

### 3.3. Animal Experiment Design

All animal experimental procedures were approved by the Experimental Animal Center, Shantou University. All animal care and experiments were carried out according to the guidelines of the Animal Ethics Committee of Shantou University (animal ethical approval protocol number: SUMC2022-036).

Eight-week-old female KM mice (*n* = 24, weight = 30 ± 3 g) were purchased from the Guangdong Medical Laboratory Animal Center (Guangdong, China). All mice were maintained under a controlled temperature (25 ± 1 °C) and humidity (60 ± 10%) with a 12 h light/dark cycle. They had free access to water and were fed with a standard mice chow diet. All mice were divided into four groups with six mice in each group. The mice were administered intragastrically with different doses of PHP (0, 75, 150, and 300 mg/kg body weight of mice) for 15 days. Fresh fecal samples were collected on the final day of the experiment, frozen in liquid nitrogen, and stored at −80 °C for subsequent bacterial population analysis. All mice were euthanized at the end of the experiment. The colon tissues were collected, and each mouse’s colon length was measured.

### 3.4. Measurement of Moisture Content

The luminal contents in the colon were dried at 105 ± 2 °C using the hot oven until they reached the constant weight. The moisture content was calculated by the following formula: moisture content = (FW − DW)/FW × 100%, where FW is the initial weight of luminal content, and DW is the final weight of luminal content.

### 3.5. Measurement of pH Value

The pH values of luminal contents in the colon were measured according to the previously reported method [28]. Briefly, the luminal contents were diluted with distilled water at a ratio of 1:10 (*w/v*). Then the pH values were determined by a digital pH meter.

### 3.6. Measurement of Colon Length and Colon Index

After removing the luminal contents from the colon of each mouse, the colon lengths were weighed. The colon indexes were measured by colon weights divided by the body weights of mice.

### 3.7. Determination of SCFAs

The fecal SCFAs were determined using gas chromatography according to our previous method [12]. In brief, 100 mg of fecal samples were dissolved in water at a ratio of 1:9 (*w/v*). After centrifugation (12,000× *g*, 4 °C, 10 min), the supernatants were filtered using 0.45 μm membranes and mixed with 0.03 mL of 2-ethlbutyric acid (0.04 M) and 0.02 mL of HCL (6 M). Then, the supernatant of the mixtures was analyzed for the levels of SCFAs through gas chromatography (GC-2010 Plus system, Shimadzu, Japan) that had a DB-WAXetr column (30 cm × 0.25 mm × 0.25 μm, Agilent Technologies, Stockport, UK) and a flame ionization detector. Standard calibrations were used to estimate the contents of SCFAs by determining the peak areas.

### 3.8. Histological Analysis

The colon samples were cut into small pieces, treated with 10% formalin solution for fixation, and subsequently embedded in paraffin. The sections with a thickness of 4 μm were prepared and stained with H&E or AB&PAS. The stained sections were observed under a light microscope (OLYMPUS, CH20, Tokyo, Japan) and photographed (magnification 50×). The morphological structure of the colon and mucosal thickness were analyzed. The number of goblet cells and AB-PAS positive cells were quantified by examining at least 100 epithelial cells in five distinct intestinal villi of every mouse section [28].

### 3.9. Immunohistochemical Analysis

The histological sections were subjected to deparaffinization with xylene, followed by rehydration using a graded ethanol series. For antigen retrieval, the sections were heated in 0.01 M sodium citrate buffer (pH 6.0) at 95 °C for 20 min. After treatment with 5% goat serum in PBS (0.1 M, pH 7.2) to block nonspecific protein binding, the mucin (MUC-2), tight proteins (ZO-1 and occludin), an inflammatory protein (MPO) was detected using primary antibodies specific to it, which were subsequently bound with a secondary IgG antibody conjugated to HRP. The distribution and expression of proteins including MUC-2, ZO-1, occludin, and MPO were observed under light microscopy (OLYMPUS, CH20, Tokyo, Japan) and photographed (magnification 50×). The MUC-2, ZO-1, occludin, and MPO-positive areas were analyzed using Image J software (version 1.49, National Institutes of Health, Bethesda, MD, USA).

### 3.10. DNA Extraction, 16S rRNA Amplification, and Sequencing

Fecal samples of mice were collected and dissolved in distilled water. After centrifugation (12,000× *g*, 4 °C, 10 min), the g QIA AMP DNA Stool Mini Kit (Qiagen, Shanghai, China) was used to extract genomic DNA from the fecal samples. Then the V4 region of the 16S rRNA gene was amplified using 338F forward and 518R reverse primers. All PCR reactions were performed using the Biometra PCR system (Gottingen, Germany). Subsequently, the PCR products were purified using the Poly-Gel Extraction Kit (Omega Engineering, Inc, Norwalk, CT, USA). Furthermore, the purified amplicons were sequenced by the Illumina HiSeq 2500 sequencing platform.

### 3.11. Bioinformatic Analysis of Gut Microbiota

After cutting off the barcode and primer sequence, the raw tags were obtained using fast length adjustment of short reads to improve genome assemblies (FLASH v1.2.11). Then the clean tags were produced through the Quantitative Insights into Microbial Ecology (QIIME v1.9.1) quality control process. In addition, the chimera sequences of clean tags were detected using the UCHIME algorithm compared with the reference database. Finally, effective tags were obtained by removing the chimera sequences. Sequence with ≥97% similarity of the same operational taxonomic units (OTUs) was used to calculate the richness and diversity indices of the intestinal bacterial community and the relative abundance in fecal samples. The alpha diversity index, including Chao1, Shannon, Simpson, and ACE, and the beta diversity index (PCoA analysis) were calculated using the QIIME software. The Galaxy Online Analysis Platform was used to apply the linear discriminant analysis (LDA) and LDA effect size (LefSe) methods for analyzing the metagenomic biomarker among groups, with a logarithmic score threshold of 4.0.

### 3.12. Statistical Analysis

The data are presented as mean ± SEM of three independent experiments. The statistical significance was analyzed by one-way ANOVA followed by Tukey’s test (* *p* < 0.05, ** *p* < 0.01, and *** *p* < 0.001) using GraphPad Prism (V6.0, La Jolla, CA, USA).

## 4. Conclusions

In conclusion, this study suggested that PHP had beneficial effects on the gut microbiota and intestinal barrier function. Oral administration of PHP improved intestinal physiological status and fortified the intestinal mucus layer thickness. PHP increased the moisture content and lowered the pH environment in the colon, which promoted the growth of beneficial bacteria and increased the production of SCFAs. PHP improved the structure and function of the intestinal barrier by increasing the mucin-producing goblet cells and the expression of MUC-2, ZO-1, and occludin. Furthermore, PHP increased the richness and diversity of gut microbiota and the ratio of Firmicutes to Bacteroidetes in mice. PHP regulated the composition of the gut microbiota by increasing the abundance of beneficial bacteria such as proteobacteria while decreasing the abundance of potentially harmful bacteria such as *Bacteroidaceae*. These findings revealed that PHP may have the potential as a dietary supplement to promote gut health and prevent various health conditions related to gut dysbiosis and intestinal barrier dysfunction. Further research is needed to investigate the potential therapeutic effects of PHP on intestinal inflammation and other intestinal diseases.

## Data Availability

The data that support the findings of this study are available from the corresponding author upon reasonable request.
